# Enhancing Predictive Accuracy for Recurrence-Free Survival in Head and Neck Tumor: A Comparative Study of Weighted Fusion Radiomic Analysis

**DOI:** 10.3390/diagnostics14182038

**Published:** 2024-09-14

**Authors:** Mohammed A. Mahdi, Shahanawaj Ahamad, Sawsan A. Saad, Alaa Dafhalla, Alawi Alqushaibi, Rizwan Qureshi

**Affiliations:** 1 Information and Computer Science Department, College of Computer Science and Engineering, University of Ha’il, Ha’il 55476, Saudi Arabia; m.mahdi@uoh.edu.sa; 2 Software Engineering Department, College of Computer Science and Engineering, University of Ha’il, Ha’il 55476, Saudi Arabia; s.ahamad@uoh.edu.sa; 3 Computer Engineering Department, College of Computer Science and Engineering, University of Ha’il, Ha’il 55476, Saudi Arabia; sa.saad@uoh.edu.sa (S.A.S.); a.dafhalla@uoh.edu.sa (A.D.); 4 Computer and Information Sciences, Universiti Teknologi PETRONAS, Seri Iskandar 32610, Malaysia; alawi_18000555@utp.edu.my; 5 Fast School of Computing, National University of Computer and Emerging Sciences, Karachi 75270, Pakistan

**Keywords:** head and neck cancer, recurrence-free survival, radiomics, weighted fusion, predictive modeling

## Abstract

Despite advancements in oncology, predicting recurrence-free survival (RFS) in head and neck (H&N) cancer remains challenging due to the heterogeneity of tumor biology and treatment responses. This study aims to address the research gap in the prognostic efficacy of traditional clinical predictors versus advanced radiomics features and to explore the potential of weighted fusion techniques for enhancing RFS prediction. We utilized clinical data, radiomic features from CT and PET scans, and various weighted fusion algorithms to stratify patients into low- and high-risk groups for RFS. The predictive performance of each model was evaluated using Kaplan–Meier survival analysis, and the significance of differences in RFS rates was assessed using confidence interval (CI) tests. The weighted fusion model with a 90% emphasis on PET features significantly outperformed individual modalities, yielding the highest C-index. Additionally, the incorporation of contextual information by varying peritumoral radii did not substantially improve prediction accuracy. While the clinical model and the radiomics model, individually, did not achieve statistical significance in survival differentiation, the combined feature set showed improved performance. The integration of radiomic features with clinical data through weighted fusion algorithms enhances the predictive accuracy of RFS outcomes in head and neck cancer. Our findings suggest that the utilization of multi-modal data helps in developing more reliable predictive models and underscore the potential of PET imaging in refining prognostic assessments. This study propels the discussion forward, indicating a pivotal step toward the adoption of precision medicine in cancer care.

## 1. Introduction

The predictive modeling of tumor outcomes in patients with head and neck cancer utilizing imaging data stands as a pivotal tool for the advancement in personalized medicine [1,2,3]. Positron Emission Tomography (PET) and Computed Tomography (CT) scans, owing to their complementary strengths, offer a comprehensive view of the tumor’s metabolic activity and anatomical structure [4,5], respectively. Therefore, effectively leveraging these dual modalities for improved prediction outcomes remains a challenging frontier in medical image analysis [6]. Traditional approaches often treat the modalities separately or concatenate the data at an early stage, potentially overlooking the nuanced, modality-specific features critical for accurate prediction [7,8,9]. On the other hand, the notable frequency of loco-regional recurrences in head and neck cancers post-radiotherapy poses a significant challenge [10]. Various patient-related clinical information and tumor conditions have been identified as prognostic factors critical for determining recurrence-free survival (RFS) [11]. However, selecting these factors relevant to RFS is a meticulous and time-intensive task [12]. This underscores the critical need for devising an automated model capable of swiftly predicting and analyzing patient-specific RFS, thereby streamlining the process and enhancing the efficiency of prognostic evaluations.

Recent developments in deep learning, particularly the transformer architecture, have shown exceptional promise in handling complex, sequential data across various domains [13,14,15,16]. Despite significant progress, the heterogeneity of tumor biology and the multifaceted nature of oncological responses pose substantial challenges in prognostication [17,18]. Traditional clinical predictors, while foundational, often fall short in capturing the complex interplay of factors influencing cancer recurrence [19]. Conversely, the advent of radiomics has ushered in a new era of precision medicine [20], enabling the extraction of high-dimensional data from medical images to elucidate tumor characteristics with unprecedented detail [21].

However, the integration of radiomic features with clinical data remains an underexplored frontier, particularly in the context of head and neck cancer [22]. This gap underscores a pressing need for innovative approaches that can harness the full potential of multimodal data to enhance RFS prediction. Furthermore, the advent of Automated Machine Learning (AutoML) methods, exemplified by AutoGluon, offers a promising solution to this challenge, automating complex data processing and model selection tasks to optimize predictive performance [23,24]. This study aims to bridge these gaps by evaluating the efficacy of different data fusion techniques, including weighted fusion approaches and the incorporation of peritumoral contextual information, in improving RFS prediction for H&N cancer. Also, investigating the advantages of features fusion at the image level and the feature fusion level, utilizing a comprehensive dataset comprising clinical parameters and radiomic features extracted from CT and PET scans, we explore the prognostic value of integrating these diverse data sources. 

By leveraging AutoML capabilities, we aim not only to enhance the precision of RFS predictions but also to illuminate the comparative advantages of feature-level versus image-level data fusion in oncological prognostication. Our findings reveal the significant prognostic potential of this integrated approach, which not only surpasses the predictive accuracy of individual modalities but also underscores the importance of selecting optimal fusion strategies to maximize prognostic efficacy. 

This paper is organized as follows. The literature review in Section 2 offers a critical examination of prior work, Section 3 is dedicated to methods and materials, and Section 4, presents results and a detailed discussion. The paper concludes with Section 5, where we reflect on the implications of our work for the field of medical imaging and predictive oncology, highlighting this study’s contributions and proposing directions for future research.

## 2. Related Works

The prognostic assessment of RFS in H&N cancer has garnered significant attention in oncological research, due to its critical implications for patient management and therapeutic decision-making. Recent advancements show a shift towards integrating multidimensional data sources, including clinical parameters and radiomic features, to refine predictive models. Notably, studies have increasingly focused on the potential of radiomics—the quantitative analysis of medical images—to capture tumor heterogeneity and improve prognostic accuracy. For instance, Wang et al. [25] utilized a conventional radiomics approach, leveraging nnU-Net for segmentation and extracting radiomic features from both PET and CT images using PyRadiomics package. Their methodology involved using a single mask for both primary and nodal tumor volumes, selecting features through univariate analysis and correlation to reduce redundancy, and employing Cox Proportional Hazard models with 5-fold cross-validation across different data inputs (clinical, PET, and CT) and their combined risk scores. Clinical variables were uniquely handled by coding missing values as a separate category rather than imputing them. The integrative model, which combined risk scores from clinical, PET, and CT data, achieved a C-index of 0.67 on the test dataset. 

Similarly, Xu et al. [26] adopted a machine learning strategy for feature extraction, focusing on conventional metrics and radiomic features, with their model attaining a C-index of 0.658.

Müller et al. [27] expanded on the “Deep Fusion V2” methodology by integrating a Convolutional Neural Network (CNN) for deep feature extraction with a Multi-Layer Perceptron (MLP) for survival analysis, showing superior performance on the validation set. Thambawita et al. explored various prognostic modeling strategies, with their third approach incorporating clinical variables and image data through XGBoost, achieving a C-index of 0.656.

Thambawita et al. [28] explored two initial methodologies for prognostic modeling: the first approach utilized solely clinical data, while the second combined clinical variables with fundamental segmentation-derived features, namely the volume and the z-extent, employing a random forest that concentrated on segmentation and evaluated the prognostic significance of features derived from segmentation masks, achieving a C-index of 0.627. Wang et al. implemented a ResNet architecture for predicting RFS, with the PET-only model achieving the highest C-index of 0.70.

Salahuddin et al.’s [29] research predominantly concentrated on the task of segmentation. However, they also assessed the prognostic significance of several features derived from the segmentation masks. This evaluation included the largest volumes of tumors and lymph nodes, as well as the number of lymph nodes, using a 5-fold cross-validation approach to validate their findings. By integrating these three specific features into their analysis, they achieved a C-index of 0.627 on the test set, indicating a notable level of predictive accuracy in their prognostic model.

Wang et al. [30] implemented a ResNet architecture for predicting recurrence-free survival, experimenting with images from PET only, CT only, and combined PET/CT as distinct channels. Their methodology also considered whether to include segmentation masks, generated from the first task using Retina U-Net, in their models. Employing a 3-fold cross-validation strategy to assess the performance of all possible combinations, they observed C-index values ranging between 0.64 and 0.70 across the different setups. Remarkably, the model utilizing PET images exclusively emerged as the most effective, achieving the highest C-index of 0.70. When evaluated on the test set, the performance of this PET-only model, calculated by averaging the outcomes of the three models from the 3-fold cross-validation, reached a C-index of 0.635, showcasing its considerable predictive capability in the context of RFS prediction.

## 3. Materials and Methods

This study employs a comprehensive approach to predict RFS in head and neck cancer patients, leveraging dual PET/CT imaging and advanced machine learning techniques. By integrating clinical data with radiomic features through weighted fusion algorithms, the methodology aims to enhance the accuracy of prognostic models. The following section outlines the dataset characteristics, preprocessing steps, and the detailed processes involved in feature extraction, model development, and evaluation, providing a robust framework for assessing the effectiveness of the proposed predictive models.

### 3.1. Dataset

The dataset used in this study was obtained from the HECKTOR Challenge at MICCAI 2022 [31], comprising FDG-PET/CT scans from nine distinct centers across Canada, the United States, Switzerland, and France. The dataset includes 524 cases for training and 359 cases for testing (no reference contours were provided for the test cases), specifically focusing on H&N cancer within the oropharynx region. This diverse, multi-center dataset enhances the robustness and generalizability of the predictive models developed in this study.

**Figure 1:**
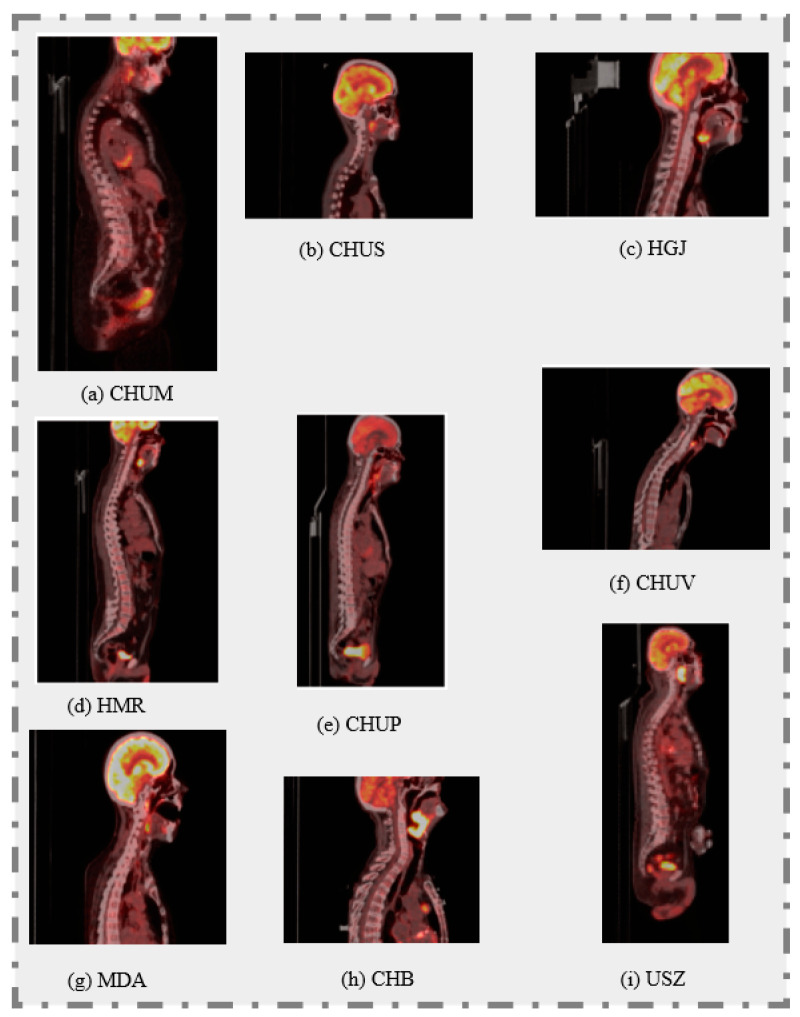
2D sagittal slices of fused PET/CT images from each of the nine participating centers.

Each center contributed varying numbers of cases (shown in Table 1), ensuring a wide range of imaging data, which is critical for training ML models to be capable of generalizing across different clinical settings. The scans were meticulously annotated by expert radiation oncologists, ensuring high-quality ground truth for tumor segmentation tasks. The dataset also includes detailed clinical follow-up data, documenting recurrence-free survival outcomes, which serve as the benchmark for evaluating the performance of the predictive models. Additionally, Figure 1 illustrates 2D sagittal slices of fused PET/CT images from each of the nine participating centers, demonstrating the variability in fields of view. The images combine CT data in grayscale (with a Hounsfield unit window of [−140, 260]) and PET data (with a Standard Uptake Value (SUV) window of [0, 12]), depicted in a “hot” colormap.

The original annotations for the training and test sets varied across centers. For example, in CHUV, CHUS, HGJ, and HMR, an expert radiation oncologist drew the GTVp and GTVn contours, with some directly on the PET/CT scan’s CT images and others on a different CT scan, later registered to the PET/CT. In CHUP, the primary tumor’s metabolic volume was initially determined using FLAB and then manually edited. In MDA, available radiotherapy contours were refined, while in USZ, tumors were segmented separately in CT and PET images, with specific handling of artifacts. The dataset from CHB involved manual drawing of GTVp and GTVn by senior nuclear medicine physicians using PET VCAR. Expert quality controls were conducted on all datasets to ensure ground-truth contour consistency.

For data preparation, experts reannotated contours to match the actual tumor volume, which was often smaller than the initially delineated radiotherapy volumes. A centralized cloud environment facilitated uniform annotation. For cases lacking original GTVp or GTVn radiotherapy contours, experts used PET/CT fusion and N staging data for annotation. Cases with PET and CT misregistration were excluded. Additionally, detailed annotation guidelines developed by the expert board were used for this quality control process. The guidelines for annotating primary tumors in PET/CT images were provided in the study [15] to participants during the challenge, and these were also adhered to in our paper. These guidelines include specific instructions for the contouring process, considering both PET and unenhanced CT acquisitions. They emphasize the importance of accurate and consistent annotation practices to ensure the reliability of the tumor segmentation process. The ground-truth data for patient outcomes, utilized as the benchmark for prediction tasks, were meticulously compiled from the clinical records documented during patient follow-ups.

### 3.2. Study Population and Design

The study population was derived from the HECKTOR 2022 challenge dataset [31], focusing on patients diagnosed with oropharyngeal cancer who underwent initial staging using FDG-PET/CT imaging. This dataset comprises cases collected from nine different centers, ensuring a diverse and representative sample of the population typically encountered in clinical practice. The inclusion and exclusion criteria and the study assessment aim are discussed in the following subsections.

#### 3.2.1. Inclusion and Exclusion Criteria

Patients included in this study were those with histologically confirmed oropharyngeal H&N cancer, who had undergone initial staging using FDG-PET/CT imaging. Only patients who had completed definitive radiotherapy, with or without concurrent chemotherapy, and had achieved complete responses to treatment were included, as this was necessary for defining RFS. This study required complete pre-treatment FDG-PET/CT imaging and the availability of key clinical data, including center, age, gender, weight, tobacco and alcohol consumption, performance status, HPV status, and treatment details. Patients were excluded from this study if they had not achieve complete responses after treatment, as their inclusion would confound the definition of RFS. Additionally, patients with missing critical clinical data that could not be reasonably estimated were excluded. For instance, weight data were missing for six training cases and two test cases; in these instances, weight was estimated at 75 kg to compute the Standard Uptake Values (SUVs) [31]. The study cohort exhibited variability in prognostic factors, including HPV statuses, treatment modalities, and other clinical parameters. While this heterogeneity reflects the real-world complexity of clinical practice, it also introduces certain challenges in model training and interpretation. Despite these limitations, the diversity of the dataset enhances the generalizability of the study findings, as reported in [31].

#### 3.2.2. Assessment Aim

The primary clinical endpoint of this study was RFS, defined as the time from the last day of radiotherapy (*t* = 0) to the reappearance of a lesion or the appearance of new lesions (local, regional, or distant). Only patients who had achieved complete responses to treatment were included in the analysis, with deaths treated as censored events to focus exclusively on RFS outcomes. The analysis utilized time-to-event data measured in days from the end of treatment to the occurrence of the event.

### 3.3. Preprocessing

The preprocessing steps were streamlined to focus on essential procedures critical for the accuracy and reproducibility of this study. Key steps included normalization of voxel intensities to standardize the dynamic range across images. Mathematically, this can involve z-score normalization, where each voxel intensity *I_xyz_* in a 3D image is transformed as

(1) $$I_{xyz}^{\prime }=\frac{I_{xyz}-\mu }{\sigma }$$

where $I_{xyz}^{\prime }\;$is the normalized intensity, μ is the mean intensity across the image volume, and σ is the standard deviation of the intensities. Contrast enhancement [32] techniques are applied to each modality to improve the visibility of critical features. For PET images, the goal is to accentuate areas of high radiotracer uptake, which are often indicative of malignancy. In the case of CT, enhancement algorithms aim to increase the clarity of anatomical structures. The transformation function for contrast enhancement can be represented as

(2) $$I^{\prime }=f(I)$$

where I is the original voxel intensity, and $I^{\prime }\;$is the enhanced intensity. The specific form of the function f depends on the enhancement technique employed (e.g., logarithmic mapping and histogram equalization). Cropping focuses the analysis on the region of interest (ROI) by removing irrelevant background and reducing computational load. The process involves selecting a sub-volume that encapsulates the tumor and adjacent anatomical landmarks critical for diagnosis and treatment planning. The cropped image *I*_crop_ is defined by spatial boundaries within the original volume *I*_original_:

(3) $$I_{\mathrm{crop}\;}=I_{\mathrm{original}\;}\left[x_{min}:x_{max},y_{min}:y_{max},z_{min}:z_{max}\right]$$

where $\left[x_{min}:x_{max},y_{min}:y_{max},z_{min}:z_{max}\right]\;$defines the 3D bounding box of the ROI. Voxel spacing homogenization was applied due to the different resolutions of PET and CT images. This process involves a resampling of the images to have consistent voxel dimensions, facilitating accurate image fusion [33] and comparison. The transformation for homogenization can be represented by

(4) $$I_{\mathrm{resampled}\;}=Resample\left(I_{\mathrm{original}\;},dX,dY,dZ\right)$$

where $I_{\mathrm{resampled}\;}\;$is the image with homogenized voxel spacing, and *dX*, *dY*, *dZ* are the desired uniform voxel dimensions. Data augmentation plays a critical role in enhancing the robustness and generalizability of the segmentation model. By applying techniques such as random cropping, where a 192 × 192 × 192 voxel patch is extracted from the H&N area centered on the foreground classes with probabilities of 0.45 for tumor, 0.45 for lymph nodes, and 0.1 for background, and flipping, we introduce variability that effectively expands the dataset. These preprocessing steps address the inherent heterogeneity in multi-modal imaging datasets, improving the quality of the input data, which is essential for the accurate, reproducible, and robust segmentation of tumors in downstream machine learning models.

### 3.4. Radiomics Feature Extraction

Radiomics involves extracting a large number of quantitative features from medical images that capture the underlying pathology, including characteristics that may not be discernible to the human eye. In this study, 2059 radiomic features were extracted from both CT and PET scans using advanced computational techniques. The extracted features included intensity-based metrics, such as histograms of voxel intensities, and shape features are computed from the delineated tumor volumes to quantify geometrical attributes. Textural features are also extracted, utilizing advanced matrices such as the Gray Level Co-occurrence Matrix (GLCM), Gray Level Dependence Matrix (GLDM), Gray Level Run Length Matrix (GLRLM), and Gray Level Size Zone Matrix (GLZSM), which serve to characterize the intricate patterns within the tumor’s internal structure.

### 3.5. Weighted Fusion of CT and PET Data

The primary objective of this work was to enhance the predictive accuracy of recurrence-free survival by employing a weighted fusion of CT and PET data. We evaluated three main approaches: CT alone, PET alone, and a combination of CT and PET data using various fusion weights. In the weighted fusion approach, different proportions of CT and PET features were combined to form integrated feature sets, with weights ranging from 10% to 90% for PET data. The optimal fusion weight was determined based on the predictive performance of the resulting models in survival analysis, with a particular focus on maximizing the concordance index (C-Index). This approach allowed us to systematically assess the contribution of each imaging modality to the prediction of RFS and to identify the most effective combination of data for this purpose.

### 3.6. Proposed Framework

**Figure 2:**
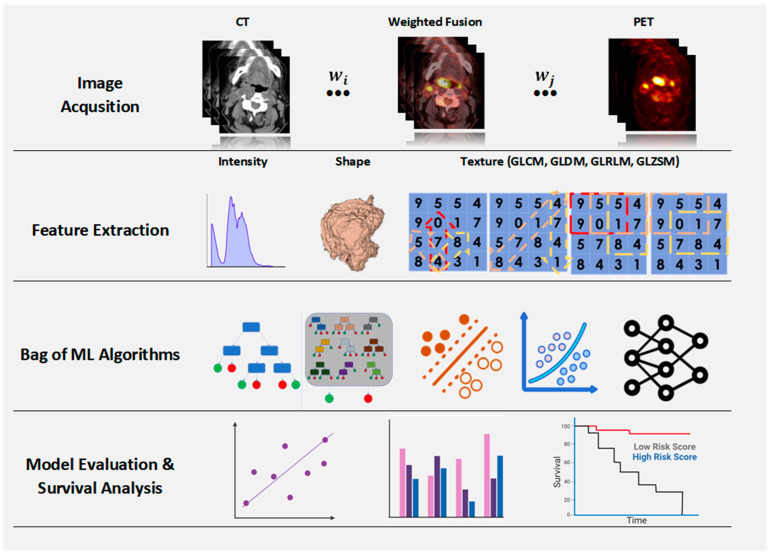
The comprehensive methodology of the Weighted Fusion Bag of ML Algorithms for dual PET/CT imaging in predicting head and neck tumor outcomes.

Figure 2 encapsulates the methodology adopted for predictive analysis of H&N tumor outcomes using dual PET/CT imaging. This multifaceted approach is structured in distinct, interconnected stages, ensuring the systematic extraction and fusion of critical imaging features for prognostic assessment. The initial stage entails the acquisition of CT and PET scans, which are pivotal in providing detailed insights into the anatomical structure and metabolic function of the tumors, respectively. This dual imaging technique lays the groundwork for a comprehensive dataset, indispensable for subsequent analysis. Following image acquisition, a rigorous feature extraction process is implemented. This involves the derivation of intensity features from the image histograms, encapsulating the distribution of pixel or voxel intensities within the scan.

At the core of the methodology lies the weighted fusion process [34], a strategic phase where features from the CT and PET modalities are integrated. This integration is not merely additive but is governed by a set of learned weights (denoted by *W_i_*) and (*W_j_*), which selectively amplify features based on their prognostic significance. This handmade fusion process is designed to capitalize on the distinctive diagnostic values of the CT and PET features, effectively synthesizing them into a potent predictive model.

### 3.7. Bag of ML Algorithms

Following the fusion, a comprehensive ensemble of ML algorithms, termed a ‘Bag of ML Algorithms’, is employed. This ensemble approach not only embraces the diversity of algorithmic strategies but also mitigates the risk of overfitting, enhancing the model’s generalizability and robustness. The algorithms are calibrated on the fused feature set, fostering a model that is adept at handling the multifaceted nature of tumor imaging data. The top-tier algorithms utilized within the AutoGluon framework [35] include Gradient Boosting Machines (GBMs) with implementations such as XGBoost, LightGBM, and CatBoost, known for their robustness in structured data. It also leverages the ensemble strengths of the random forest and Extra Trees Classifiers, renowned for their performance and ability to mitigate overfitting. The simplicity and effectiveness of K-Nearest Neighbors (KNNs) is harnessed alongside traditional algorithms like Linear and Logistic Regression, providing foundational statistical inference. Deep learning models implemented via advanced neural network frameworks provide complex pattern recognition capabilities. Furthermore, the suite integrates Support Vector Machines (SVMs) for their proficiency in high-dimensional spaces, while Bayesian Optimization is employed to fine-tune the models. AutoGluon encapsulates these algorithms within a meta-framework of stacking and ensemble techniques, automatically calibrating the weights and integration of predictions to derive a final, superior model, reflective of the intricate data landscape inherent in oncological prognostication.

The final phase of the methodology is devoted to model evaluation and survival analysis. The predictive performance is meticulously assessed using a suite of evaluation metrics, providing a multi-dimensional view of the model’s accuracy and reliability. The survival analysis visualized with Kaplan–Meier curves stratifies patients into risk categories based on the model’s predictions. These curves offer a graphical representation of the survival probability over time, distinguishing between low- and high-risk cohorts. This critical analysis underscores the model’s clinical relevance, demonstrating its potential to inform therapeutic decision-making and prognostication.

### 3.8. Performance Evaluation Metrics 

To assess the predictive performance of the models developed in this study, we employed several evaluation metrics that are widely recognized in survival analysis and machine learning. These metrics provide a comprehensive view of the model’s accuracy, robustness, and discriminatory power. Below, we describe each metric along with its corresponding formula.

#### 3.8.1. Root Mean Square Logarithmic Error (RMSLE)

RMSLE is a metric that measures the disparity between the predicted and actual survival times on a logarithmic scale. It penalizes underestimations more heavily than overestimations, making it particularly useful for survival analysis where early predictions can have significant implications.

$$RMSLE=\sqrt{\frac{1}{n}\sum_{i=1}^{n}\;\;{\left(log\left({\hat{y}}_{i}+1\right)-log\left(y_{i}+1\right)\right)}^{2}}$$

where n is the number of data points, ${\hat{y}}_{i}$ is the predicted survival time for the $i^{th}$ data point, and $y_{i}$ is the actual survival time for the $i^{th}$ data point.

#### 3.8.2. Mean Absolute Percentage Error (MAPE)

The MAPE measures the average magnitude of the errors in prediction as a percentage. This metric is useful for understanding the relative accuracy of predictions, particularly in the context of survival times that can vary significantly across patients.

$$MAPE=\frac{100\%}{n}\sum_{i=1}^{n}\;\left|\frac{y_{i}-{\hat{y}}_{i}}{y_{i}}\right|$$

#### 3.8.3. Pearson Correlation Coefficient

The Pearson correlation coefficient measures the linear relationship between the predicted and actual survival times. Values close to 1 indicate a strong positive correlation, suggesting that the model’s predictions are closely aligned with the true outcomes.

$$\rho =\frac{\sum_{i=1}^{n}\;\;\left(y_{i}-\bar{y}\right)\left({\hat{y}}_{i}-\bar{\hat{y}}\right)}{\sqrt{\sum_{i=1}^{n}\;\;{\left(y_{i}-\bar{y}\right)}^{2}\sum_{i=1}^{n}\;\;{\left({\hat{y}}_{i}-\bar{\hat{y}}\right)}^{2}}}$$

where $y_{i}$ and ${\hat{y}}_{i}$ are the actual and predicted survival times and $\bar{y}$ and $\bar{\hat{y}}$ are the mean values of the actual and predicted survival times, respectively.

#### 3.8.4. Concordance Index (C-Index)

C-Index is a metric used to evaluate the discriminatory power of survival models, particularly how well the model ranks survival times. A higher C-Index indicates better performance in distinguishing between patients with different survival outcomes.

$$C-\mathrm{Index}=\frac{1}{n_{c}}\sum_{i\neq j}\;I\left({\hat{y}}_{i}>{\hat{y}}_{j}\right)\times I\left(y_{i}>y_{j}\right)$$

where $n_{c}$ is the number of comparable pairs, I(⋅) is the indicator function, which equals 1 if the condition is true and 0 otherwise, $y_{i}$ and $y_{j}$ are the actual survival times, and ${\hat{y}}_{i}$ and ${\hat{y}}_{j}$ are the predicted survival times.

## 4. Results

In this section, we thoroughly assess the effectiveness of our proposed CT/PET weighted fusion strategy for predicting RFS in H&N cancer patients. Our evaluation focuses on comparing the predictive performance across different modalities, including CT alone, PET alone, and various fusion weights, to determine the most accurate and reliable approach for RFS outcome prediction.

### 4.1. Evaluation of Fusion Weight and Imaging Modality on RFS Prediction

Table 2 provides a comparative analysis of RFS prediction accuracies across various modalities using a bag of ML algorithms. RFS prediction is a critical measure in oncological studies, signifying the period after treatment during which the patient remains free from cancer recurrence. The table evaluates individual modalities—CT and PET scans—alongside a range of weighted fusion techniques (designated as Weighted Fusion with weights ranging from 10% to 90%) for their efficacy in RFS prediction. Table 2 provides a comparative analysis of RFS prediction accuracies across various modalities using a composite suite of ML algorithms. RFS prediction is a critical measure in oncological studies, signifying the period after treatment during which the patient remains free from cancer recurrence. The table evaluates individual modalities—CT and PET scans—alongside a range of weighted fusion techniques (designated as Weighted Fusion with weights ranging from 10% to 90%) for their efficacy in RFS prediction. 

**Figure 3:**
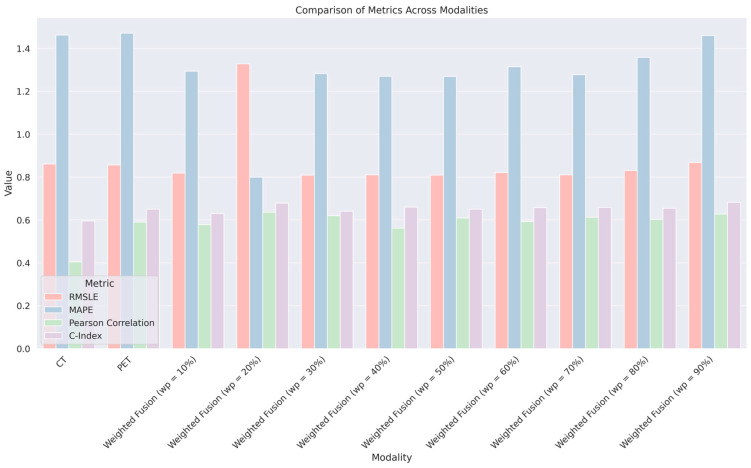
Comparison of metrics across different imaging modalities.

The evaluation is underscored by *p*-values, with a threshold of *p* < 0.001 indicating statistical significance in the observed correlations. In this context, all modalities demonstrate statistically significant predictive power. The modality offering the best RFS prediction results can be identified by analyzing the C-Index, as it directly measures the predictive model’s accuracy in distinguishing between different patient outcomes. Here, the ‘WeightedFusion (wp = 90%)’ modality achieves the highest C-Index of 0.68175, suggesting superior performance over the other modalities in predicting RFS (Figure 3). This indicates that a higher weighting (90%) in the fusion process for integrating PET and CT data leads to the most accurate RFS predictions in this study. The C-Index is complemented by the RMSLE and MAPE values, where lower scores denote better performance, and the Pearson correlation, where a value closer to one indicates a more precise prediction. The ‘WeightedFusion (wp = 90%)’ modality excels in all these metrics, corroborating its robustness as a predictive tool for RFS.

Table 3 displays an evaluation of various modality combinations and their respective performance in predicting RFS using a suite of machine learning algorithms. RFS signifies the duration during which a patient remains free from any signs of cancer recurrence post-treatment, making its prediction a pivotal aspect of oncological prognostication. All models have shown statistically significant predictive power (*p*-value < 0.001). From the results, the modality combination ‘CT_WeightedFusion (wp = 90%)’, which likely represents a weighted combination of CT imaging features with features derived from a weighted fusion approach incorporating 90% PET data, achieves the highest C-Index of 0.667177914. This suggests that this combination provides the most accurate prediction among the ones tested, balancing the unique strengths of CT and PET imaging data optimally. It is important to note that while the ‘WeightedFusion (wp = 90%)’ alone has a slightly higher C-Index than some combined modalities, the ‘CT_WeightedFusion (wp = 90%)’ combination presents a more balanced approach with a C-Index value that is competitive and suggests an improvement over using each modality in isolation. The results indicate that the integration of features from CT scans with those from a PET-dominant weighted fusion approach provides a significant enhancement in predictive accuracy for RFS outcomes. This integrative method seems to leverage the detailed anatomical data from CT scans and the metabolic information from PET scans, improving the overall prognostic power of the machine learning model.

**Figure 4:**
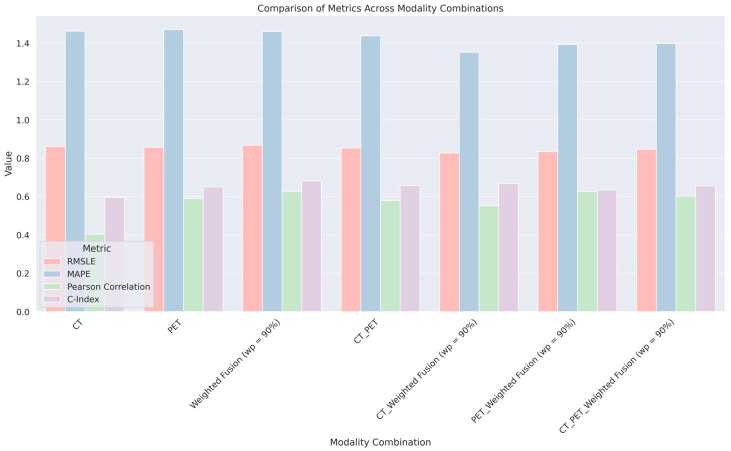
Comparison of metrics across a combination of various imaging modalities.

Figure 4 comparing different modality combinations for predicting RFS using four metrics: RMSLE, MAPE, Pearson correlation, and C-Index. The modalities analyzed include CT, PET, their direct combination (CT_PET), and several weighted fusion variations. Lower RMSLE and MAPE values indicate better accuracy, while higher Pearson correlation and C-index values denote stronger predictive performance. The chart reveals that combined modalities, particularly those using a weighted fusion approach, perform better than single imaging techniques, as evidenced by the height of the bars corresponding to each metric.

**Figure 5:**
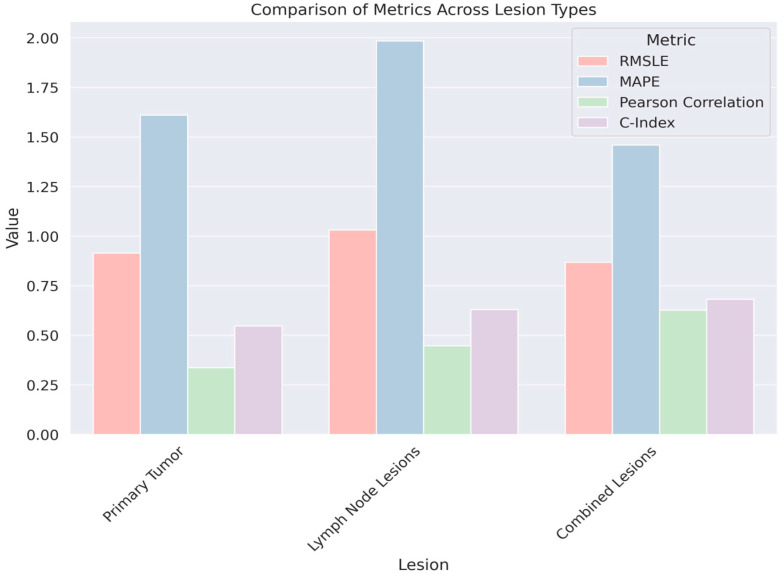
A graphical representation comparing RFS prediction across three lesion types: Primary Tumor, Lymph Node Lesions, and Combined Lesions.

Additionally, the features used in the predictive model described in Table 4 are radiomic features. Radiomics is the process of extracting a large number of quantitative features from medical images that can reflect the underlying pathology, including characteristics that may not be discernible to the human eye. In the context of the table, radiomic features from both primary tumor sites and lymph node lesions were extracted and processed using a weighted fusion approach with a strong emphasis on PET imaging features (90% weighting). The analysis indicates that the predictive model using combined radiomic features from both primary tumors and lymph node lesions (WeightedFusion (wp = 90%)) yields the most accurate RFS predictions, as evidenced by the highest C-index and Pearson correlation values, as well as the lowest RMSLE and MAPE values among the lesion types evaluated in Figure 5. This suggests that the heterogeneity of the tumor environment, captured by the comprehensive radiomic profile of both primary and associated lymph node lesions, provides a richer dataset for the model, leading to improved prognostic performance compared to models that consider radiomic features from the primary tumor or lymph node lesions in isolation.

### 4.2. Evaluation of Contextual Information Effect on on RFS Prediction

Table 5 displays the impact of incorporating contextual information from various peritumoral regions on the accuracy of RFS prediction. The contextual information is quantified by the radius (r) from the tumor boundary, with r = 0 corresponding to features extracted directly at the tumor boundary and increasing values of r representing wider peritumoral areas included in the analysis. The performance of the predictive model is quantified at different radii, assessing how the inclusion of surrounding tissue features affects the predictive accuracy.

**Figure 6:**
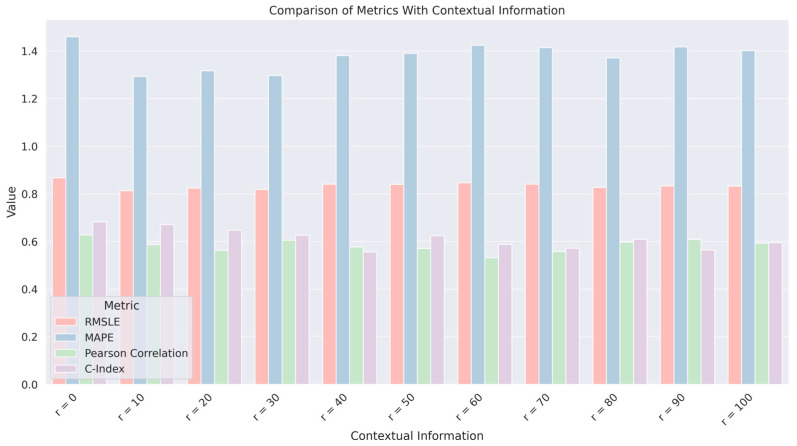
Comparative analysis of RFS prediction across various contextual distances (r = 0 to r = 100) from the tumor boundary, evaluating the impact of incorporating peritumoral tissue characteristics into the predictive modeling process.

From Table 5, the highest C-Index is observed when only the tumor boundary (r = 0) is considered for feature extraction, indicating the highest prediction accuracy without including the peritumoral context. As the radius increases, the C-Index generally decreases, suggesting that incorporating more of the surrounding tissue does not necessarily improve predictive performance and may indeed introduce noise or less relevant information. The results listed in Table 5 and Figure 6 suggest that the most relevant radiomic features for predicting RFS are located at or very close to the tumor boundary. This could indicate that the immediate interface between the tumor and surrounding tissue holds critical information for prognosis, which may be diluted when more distant regions are included.

### 4.3. Evaluation of Clinical vs. Radiomics Models on RFS Prediction

Table 6 presents a comparative analysis of RFS prediction performance using three different feature sets: clinical, radiomics, and a combination of both. Performance metrics are employed to evaluate the effectiveness of each feature set. Clinical features alone show the least predictive power with the highest RMSLE and MAPE values and the lowest Pearson correlation and C-index. This indicates lower accuracy and a weaker correlation between the predicted outcomes and actual outcomes when only clinical data are used. Radiomics features significantly improve prediction performance over clinical features alone, as evidenced by lower RMSLE and MAPE values, a higher Pearson correlation, and an improved C-Index. This suggests that imaging features provide a more nuanced understanding of the disease, which enhances the prediction of RFS. Combined features, integrating both clinical and radiomics data, yield the best performance with the lowest RMSLE and MAPE, and the highest Pearson correlation and C-index. This illustrates the advantage of a multi-modal approach that harnesses both the rich, descriptive power of radiomics and the fundamental insights provided by clinical data. The statistical significance of these predictions is affirmed by *p*-values well below the 0.001 threshold.

**Figure 7:**
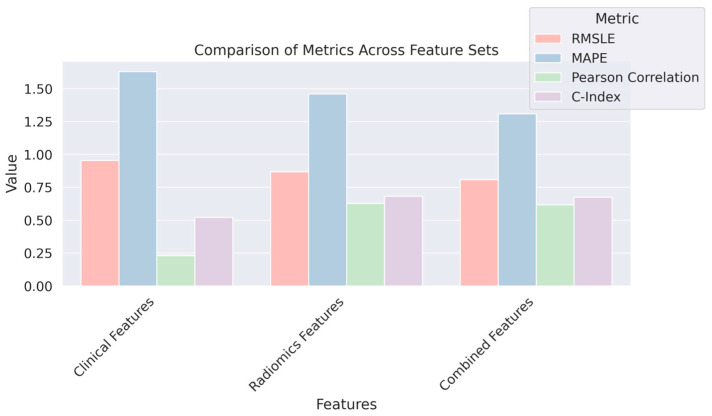
Comparison of RFS prediction accuracy using different feature sets: Clinical, Radiomics, and Combined.

Figure 7 demonstrates that lower RMSLE and MAPE values suggest better accuracy, whereas higher Pearson correlation and C-index values indicate stronger predictive ability. The Combined feature set appears to perform best, demonstrating the value of integrating clinical and radiomics data in predicting RFS.

## 5. Survival Analysis

In this study, we employed survival analysis to assess the impact of various clinical and radiomic features on RFS in patients with oropharyngeal H&N cancer. Survival analysis was conducted using the Kaplan–Meier method, which is a non-parametric statistic commonly used to estimate the survival function from lifetime data. This method allows us to visualize the survival probability over time for different patient groups and is particularly useful in comparing the survival experiences between low-risk and high-risk groups.

**Figure 8:**
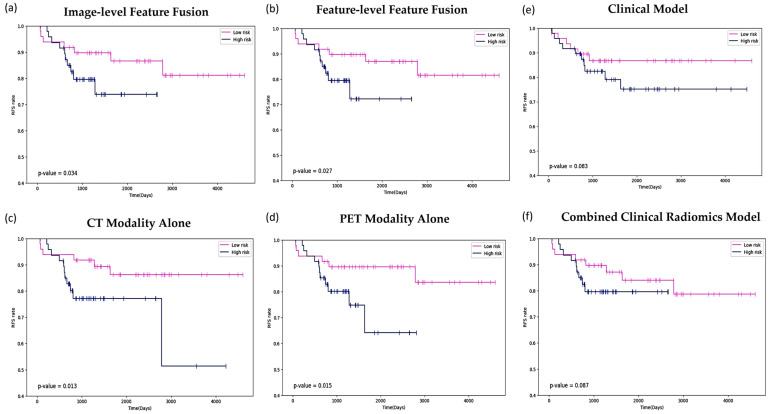
Kaplan–Meier survival curves for RFS across different model approaches. (**a**) Image-level feature fusion, (**b**) feature-level feature fusion, (**c**) CT modality alone, (**d**) PET modality alone, (**e**) clinical model, and (**f**) combined clinical and radiomics model. The *p*-values indicate the statistical significance of the differences between the low-risk and high-risk groups.

Figure 8 presents Kaplan–Meier survival curves, which are used to estimate the survival experience of patient groups over time, based on six different approaches/models (image-level feature fusion, feature-level feature fusion, CT modality alone, PET modality alone, clinical model, and combined clinical and radiomics model) applied in the analysis of RFS prediction. For the image-level fusion (Figure 8a, *p*-value = 0.034), the survival curves demonstrate a statistically significant difference between the low-risk and high-risk patient groups, with a clear divergence between the two curves. The statistical significance is supported by the *p*-value, which is lower than the 0.05 threshold. In contrast, the feature-level fusion (Figure 8b, *p*-value = 0.027) shows a more pronounced separation between the low-risk and high-risk groups. The even lower *p*-value here indicates a stronger statistical significance in the difference between the two groups’ survival experiences. Considering the *p*-values alone, the feature-level fusion approach has a stronger statistical significance in differentiating between the low- and high-risk groups compared to the image-level fusion. 

Figure 8c, d illustrate the Kaplan–Meier survival curves for RFS in patients, stratified by risk groups using CT and PET imaging modalities, respectively. Figure 8c demonstrates that CT-derived features provide a clear distinction in survival outcomes between low-risk and high-risk patients, with a *p*-value of 0.013, underscoring the significant prognostic value of anatomical information from CT scans. Similarly, Figure 8d shows that PET-derived features, which capture metabolic activity, also significantly stratify patient risk, as indicated by the *p*-value of 0.015. The similar *p*-values in both figures suggest that while each modality independently offers valuable prognostic insights, their unique contributions might be complementary in capturing different aspects of tumor biology, further justifying the exploration of their combined use in predictive modeling.

Figure 8e displays the survival curve generated by the clinical model, which stratifies patients into low- and high-risk groups based on clinical data. The *p*-value of 0.083 indicates that the observed difference in survival outcomes between the groups does not reach statistical significance, suggesting that clinical features alone may be insufficient for robust risk stratification. Similarly, Figure 8f shows the survival curve derived from the radiomics model, which uses quantitative imaging features. With a *p*-value of 0.087, this model also fails to achieve statistical significance, highlighting its limitations in independently distinguishing between survival probabilities. The close *p*-values in both models suggest that neither clinical nor radiomics data alone provide a statistically strong basis for patient stratification, emphasizing the need for more integrative approaches.

Concluding our discussion, this investigation into leveraging AutoML for predicting RFS in H&N cancer aligns with and extends the current literature on the use of advanced computational techniques in oncology. By integrating radiomics features and employing novel weighted fusion techniques at both image and feature levels, we enhanced the predictive power of our models. The image-level fusion approach successfully captures the broader tumor environment by combining various imaging modalities before feature extraction, while the feature-level fusion leverages distinct contributions from each modality, creating a more comprehensive and informative feature set.

Our findings underscore the value of a dual-fusion strategy, which when combined with the automation and efficiency of AutoML, presents a robust and multifaceted method for improving prognostic accuracy. Theoretically, this work highlights the importance of utilizing data at multiple levels—from the macroscopic imaging scale to the microscopic radiomic features—deepening our understanding of tumor biology through more detailed analyses.

Practically, the development of these sophisticated prognostic models not only enhances accuracy but also demonstrates resilience, offering practical benefits for clinical decision-making and personalized treatment planning. As the field of oncology increasingly moves towards precision medicine, the synergy between advanced computational techniques and nuanced radiomic analysis provides a promising avenue for more personalized, effective patient care. This research sets a new benchmark for future studies, showcasing the transformative potential of integrating machine learning with medical imaging to achieve a more detailed, patient-centric approach to cancer prognosis and treatment.

## 6. Conclusions

In this study, we developed a predictive model for RFS in H&N cancer patients by examining various modalities including clinical data, radiomics, and a series of weighted fusion techniques. The integration of CT and PET imaging features through weighted fusion emerged as a key strategy in enhancing predictive accuracy. Specifically, a fusion approach with a 90% emphasis on PET imaging features proved to be particularly effective, yielding the highest C-index among the techniques assessed, thereby highlighting the superior prognostic value of PET-centric features when combined with CT-derived data. However, several limitations must be acknowledged. This study’s internal validity may be affected by the inherent heterogeneity of the dataset, including variability in clinical and imaging data across different centers. While efforts were made to standardize data processing and analysis, potential biases related to data collection and preprocessing cannot be entirely ruled out. Externally, the generalizability of the study results is limited by the dataset’s specific characteristics, which may not fully represent the broader population of H&N cancer patients. The findings are particularly relevant to patients similar to those included in the MICCAI 2022 HECKTOR dataset, and caution should be exercised when extrapolating these results to different patient populations or clinical settings. Furthermore, while this study employed correlation tests to explore associations between variables, it is important to emphasize that correlation does not imply causation. The observed correlations provide insights into potential relationships, but they do not establish definitive causal links between the variables and RFS outcomes. This highlights the need for further research, potentially involving more sophisticated causal inference methods, to better understand the underlying mechanisms driving these associations. Finally, while this study has made significant strides in enhancing RFS prediction through multi-modal data integration, it also underscores the challenges inherent in this task. Future research should aim to address these limitations by incorporating larger and more diverse datasets, exploring additional prognostic variables, and employing advanced ML algorithms to refine predictive models.

## Figures and Tables

**Table 1:** Distribution of PET/CT Tumor Segmentation Multi-Center Dataset Across Participating.

No	Center	Split	#Cases
1	CHUM: Centre Hospitalier de l’Université de Montréal, Montréal, CA	Train	56
2	CHUS: Centre Hospitalier Universitaire de Sherbooke, Sherbrooke, CA	Train	72
3	HGJ: Hôpital Général Juif, Montréal, CA	Train	55
4	HMR: Hôpital Maisonneuve-Rosemont, Montréal, CA	Train	18
5	CHUP: Centre Hospitalier Universitaire Poitiers, FR	Train	72
6	CHUV: Centre Hospitalier Universitaire Vaudois, CH	Train	53
	Total	Train	524
7	CHB: Centre Henri Becquerel, FR	Test	58
8	USZ: Universitätsspital Zürich, SW	Test	101
9	MDA: MD Anderson Cancer Center, US	Test	200
	Total	Test	359

**Table 2:** Performance Metrics of different modalities for RFS Prediction.

Modality	RMSLE	MAPE	Pearson Correlation	*p*-Value	C-Index
CT	0.861	1.462	0.403	<0.001	0.595
PET	0.856	1.470	0.589	<0.001	0.650
Weighted Fusion (wp = 10%)	0.818	1.293	0.577	<0.001	0.628
WeightedFusion (wp = 20%)	1.327	0.799	0.635	<0.001	0.677
WeightedFusion (wp = 30%)	0.808	1.282	0.619	<0.001	0.640
WeightedFusion (wp = 40%)	0.810	1.269	0.561	<0.001	0.659
WeightedFusion (wp = 50%)	0.808	1.268	0.609	<0.001	0.650
WeightedFusion (wp = 60%)	0.820	1.314	0.592	<0.001	0.657
WeightedFusion (wp = 70%)	0.810	1.277	0.612	<0.001	0.657
WeightedFusion (wp = 80%)	0.830	1.358	0.602	<0.001	0.654
WeightedFusion (wp = 90%)	**0.867**	**1.460**	**0.627**	**<0.001**	**0.681**

The bold indicates that weighted fusion of 90% achieved the highest performance.

**Table 3:** Model outcomes for modality combination for RFS Prediction.

Modality Combination	RMSLE	MAPE	Pearson Correlation	*p*-Value	C-Index
CT	0.861	1.462	0.403	<0.001	0.595
PET	0.856	1.470	0.589	<0.001	0.650
**WeightedFusion (wp = 90%)**	**0.867**	**1.460**	**0.626**	**<0.001**	**0.681**
CT_PET	0.853	1.437	0.579	<0.001	0.657
CT_WeightedFusion (wp = 90%)	0.827	1.352	0.552	<0.001	0.667
PET_WeightedFusion (wp = 90%)	0.834	1.392	0.625	<0.001	0.634
CT_PET_WeightedFusion (wp = 90%)	0.846	1.397	0.601	<0.001	0.655

The bold font indicates that weighted fusion (wp = 90%) modality combination achieved the highest performance.

**Table 4:** Comparative analysis of RFS prediction using radiomic features extracted from primary tumor, lymph node lesions, and their combination.

Lesion	RMSLE	MAPE	Pearson Correlation	*p*-Value	C-Index
Primary Tumor (WeightedFusion (wp = 90%))	0.914	1.610	0.336	<0.001	0.547
Lymph Node Lesions (WeightedFusion (wp = 90%))	1.031	1.984	0.446	<0.001	0.629
Combined Lesions (WeightedFusion (wp = 90%))	**0.867**	**1.460**	**0.626**	**<0.001**	**0.681**

The bold font indicates that combined lesions weighted fusion (wp = 90%) achieved the highest performance.

**Table 5:** Performance metrics for RFS prediction using radiomic features from varying contextual information from the tumor boundary.

Contextual Information	RMSLE	MAPE	Pearson Correlation	*p*-Value	C-Index
r = 0 (Tumor Boundary)	**0.867**	**1.460**	**0.626**	**<0.001**	**0.682**
r = 10	0.813	1.292	0.586	<0.001	0.671
r = 20	0.823	1.316	0.562	<0.001	0.647
r = 30	0.817	1.296	0.604	<0.001	0.6257
r = 40	0.840	1.380	0.577	<0.001	0.556
r = 50	0.839	1.389	0.570	<0.001	0.6234
r = 60	0.845	1.423	0.531	<0.001	0.587
r = 70	0.840	1.413	0.557	<0.001	0.571
r = 80	0.826	1.370	0.597	<0.001	0.608
r = 90	0.833	1.417	0.608	<0.001	0.563
r = 100	0.832	1.401	0.592	<0.001	0.594

The bold indicates that contextual information r = 0 (tumor boundary) achieved the highest performance.

**Table 6:** Evaluation of RFS prediction accuracy using models: Clinical model, Radiomics model, and a Combined Features model.

Features	RMSLE	MAPE	Pearson Correlation	*p*-Value	C-Index
Clinical Model	0.953	1.628	0.230	0.0229	0.521
Radiomics Model	0.867	1.460	0.626	<0.001	0.681
Combined Model	0.807	1.307	0.617	<0.001	0.674

## Data Availability

The data used in this study are available and can be accessed at the HECKTOR 2022 challenge website [https://hecktor.grand-challenge.org/Data/ (24 May 2023)] upon request. This dataset includes fully anonymized FDG-PET/CT scans and associated clinical information used for the analysis in this study.
